# Metamorphosis of an identified serotonergic neuron in the *Drosophila *olfactory system

**DOI:** 10.1186/1749-8104-2-20

**Published:** 2007-10-24

**Authors:** Bidisha Roy, Ajeet P Singh, Chetak Shetty, Varun Chaudhary, Annemarie North, Matthias Landgraf, K VijayRaghavan, Veronica Rodrigues

**Affiliations:** 1 National Centre for Biological Sciences, TIFR, GKVK PO, Bangalore 560065, India; 2 Department of Biological Sciences, Tata Institute of Fundamental Research, Homi Bhabha Rd, Mumbai 400005, India; 3 Department of Zoology, University of Cambridge, Downing Street, Cambridge CB2 3EJ, UK

## Abstract

**Background:**

Odors are detected by sensory neurons that carry information to the olfactory lobe where they connect to projection neurons and local interneurons in glomeruli: anatomically well-characterized structures that collect, integrate and relay information to higher centers. Recent studies have revealed that the sensitivity of such networks can be modulated by wide-field feedback neurons. The connectivity and function of such feedback neurons are themselves subject to alteration by external cues, such as hormones, stress, or experience. Very little is known about how this class of central neurons changes its anatomical properties to perform functions in altered developmental contexts. A mechanistic understanding of how central neurons change their anatomy to meet new functional requirements will benefit greatly from the establishment of a model preparation where cellular and molecular changes can be examined in an identified central neuron.

**Results:**

In this study, we examine a wide-field serotonergic neuron in the *Drosophila *olfactory pathway and map the dramatic changes that it undergoes from larva to adult. We show that expression of a dominant-negative form of the ecdysterone receptor prevents remodeling. We further use different transgenic constructs to silence neuronal activity and report defects in the morphology of the adult-specific dendritic trees. The branching of the presynaptic axonal arbors is regulated by mechanisms that affect axon growth and retrograde transport. The neuron develops its normal morphology in the absence of sensory input to the antennal lobe, or of the mushroom bodies. However, ablation of its presumptive postsynaptic partners, the projection neurons and/or local interneurons, affects the growth and branching of terminal arbors.

**Conclusion:**

Our studies establish a cellular system for studying remodeling of a central neuromodulatory feedback neuron and also identify key elements in this process. Understanding the morphogenesis of such neurons, which have been shown in other systems to modulate the sensitivity and directionality of response to odors, links anatomy to the development of olfactory behavior.

## Background

The spatial representation of olfactory stimuli in *Drosophila *is achieved by the highly specific connectivity between neural elements within the antennal lobe. The architecture of individual components of the olfactory pathway are well defined and mechanisms specifying their development are beginning to be understood [1]. Olfactory receptor neurons (ORNs) map, in a receptor-specific manner, from the antenna to glomeruli within the antennal lobes [2,3]. Here they synapse onto projection interneurons (PNs) that, in turn, wire to dendritic fields of the mushroom bodies and the lateral horn of the protocerebrum [4]. Local interneurons (LNs), which are either GABAergic (inhibitory) or cholinergic (excitatory), arborize extensively between glomeruli, providing a substrate for lateral interactions between different information channels [5,6].

Earlier functional imaging experiments demonstrated that the odor-response spectra from ORNs and PNs are similar, suggesting, at least at a first approximation, that the antennal lobe acts 'merely' as a relay station for processing of olfactory information without significant processing [7,8]. Whole-cell recordings from the PNs, however, demonstrated that these cells have more complex responses than their primary sensory afferents, indicating that the output neurons integrate information across different populations of ORNs [9]. Further evidence for the role of lateral excitation within the circuit was obtained by silencing all ORNs wiring to a single glomerulus and recording stimulus-evoked activity from the corresponding PNs [10]. These systematic studies suggest the presence of crosstalk that couples most, if not all, glomeruli to each other. Another elegant demonstration of a high level of information processing within the primary center comes from olfactory conditioning experiments that result in the modification of the odor-evoked activity of the PNs [11]. The multi-glomerular-innervating LNs are obvious candidates for shaping input information by a combination of excitatory and inhibitory lateral interactions. Further cellular computation could be achieved through the activity of wide-field feedback neurons that connect the higher centers with the antennal lobes [12,13].

**Figure 1:**
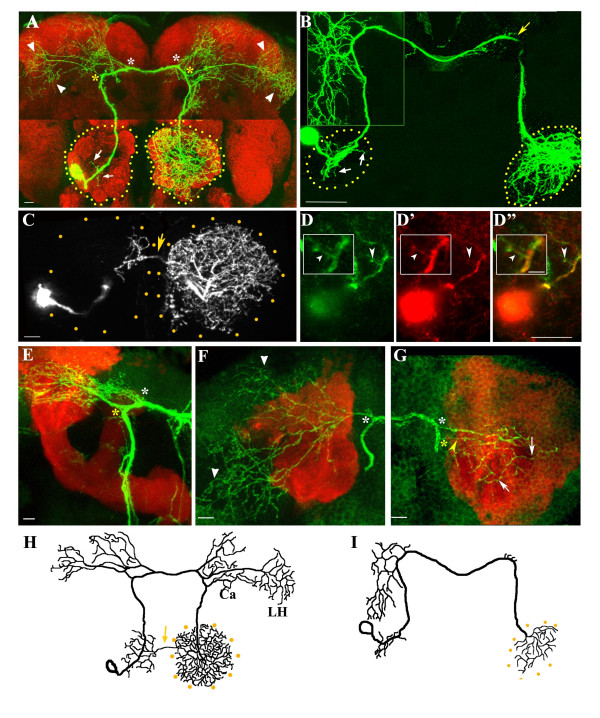
Architecture of the CSD neuron. The neuron is marked by a flipout in the RN2-*Flp*, *Tub*-FRT-CD2-FRTGal4, UAS-*mCD8GFP *strain, resulting in GFP expression in a single CSD neuron. **(a) **Only one of the pair of CSD neurons is labeled with antibodies to GFP (green) and the brain neuropile is stained with mAbnc82 (red). 1 μm sections at appropriate levels are stacked to visualize the cell body and ipsilateral dendrites (arrows), the branches to the higher centers (asterisks), projections to the lateral protocerebrum (arrowheads) in both hemispheres, and the presynaptic terminals in the contralateral antennal lobe (demarcated by yellow dots). **(b) **A single CSDn visualized in the brain of the third instar larva. Dendritic arbors ramify in the ipsilateral lobe (white arrows), branch extensively at the ipsilateral higher centers (inset) and send only a few arbors on the contralateral side (yellow arrow). The terminal arbors are extensive within the contralateral antennal lobe (demarcated with dotted lines). **(c) **In the adult, the terminal arbors of the CSDn arborize within the contralateral lobe and also send a branch in the antennal commissure (yellow arrow) to the ipsilateral antennal lobe. **(d-d") **Nod::lacZ localization in the ipsilateral dendrites (arrowheads) is visualized by anti-β-galactosidase staining (d', d", red). Inset in (d-d") shows Nod::lacZ localization in an ipsilateral neurite (arrowhead). **(e-g) **RN2-*Flp, Tub*-FRT-CD2-FRTGal4, UAS-*mCD8GFP*/mb247-DsRed animal stained with antibodies against GFP and visualized in the green (GFP) and red (ds-red) channels of the confocal microscope. Branches exit the main trunk in both hemispheres (yellow and white asterisks) and traverse anterior to the mushroom bodies to terminate within the higher centers (arrowheads in (f)). (g) A few sections are stacked to show the arborization to the calyx from the contralateral branches (arrows); the arrowhead indicates the branchpoint to the calyx neurites. **(h, i) **Schematic diagrams of the CSD neuron showing its projection patterns in the adult (h) and the larvae (i). The arrow in (h) indicates the crossover of presynaptic terminals to the lobe ipsilateral to the soma. Ca, Calyx; LH, lateral horn. Scale bar in (d) inset = 5 μm; for all other figures = 10 μm.

In this paper, we describe a pair of contralaterally projecting serotonin-immunoreactive deutocerebral (CSD) interneurons (one per hemisphere) that have extensive ramifications in the lobe as well as in the higher order neuropile in both brain hemispheres (Figure 1). These large-field neurons have been described in a variety of insects, including *Drosophila *[14-19]. Electrophysiological recordings in the silk moth *Bombyx mori *demonstrated that the CSD neurons respond to mechanosensory stimulation [20]. Application of serotonin to an antennal lobe preparation altered the excitability of PNs and LNs to stimuli [21]. Taken together, these results suggest that mechanosensory stimulation (for example, air currents) could trigger serotonin release from the CSD neuron and set the threshold of detection of odorants. Such a circuit could have important behavioral consequences, such as described in the male silk moth, which modulates response to female pheromones [22].

We use a mosaic method coupled with confocal imaging of brain whole-mounts to study the developmental architecture of the CSD neuron. The marking method also allows the specific genetic manipulation of the neuron. The CSD neuron is born in the embryo, has a relatively simple pattern in the larval stages and is remodeled during pupation to give rise to an elaborate adult pattern. We chart the changes occurring during metamorphosis and examine which events are due to the autonomous action of the ecdysterone-receptor pathway. We test whether targeted expression of molecules that compromise vesicle recycling, neuronal activity and retrograde transport affect aspects of remodeling. Finally, we show that neurons in the mushroom bodies and sensory neurons do not affect the process of remodeling, while alteration in the number of interneurons (PNs and/or LNs), possible targets of the CSD, have an effect. Our results lay the groundwork for further analysis of how intrinsic and extrinsic cues guide remodeling of an identified central neuron.

## Results

### The CSD interneuron is specifically labeled by a flip-out technique

**Figure 2:**
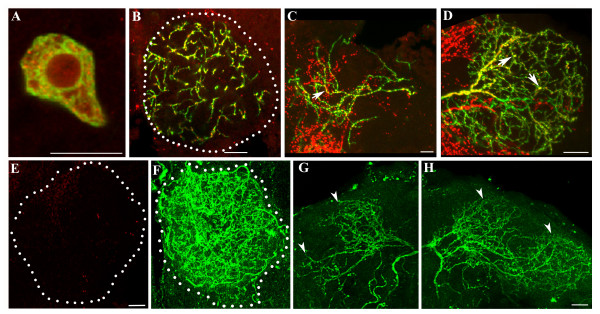
Serotonin immunoreactivity and the effect of serotonin depletion on the CSDn. **(a-d) **Staining using anti-serotonin antibodies (red) in different regions of the CSDn (green); (a) cell body, (b) contralateral terminals, (c) branches in the ipsilateral and (d) contralateral higher centers (arrows highlight puncta of serotonin immunoreactivity). **(e-h) ***ddc*^ts2^/*ddc*^ts2^; RN2-*Flp, Tub*-FRT-CD2-FRTGal4, UAS-*mCD8GFP*/+ animals grown at restrictive temperature show a strong reduction in serotonin immunoreactivity within the antennal lobe (e). The presynaptic terminals from a single CSDn (f), as well as the ipsilateral (g) and contralateral (h) arbors over the higher centers (arrowheads) do not show any appreciable change in branching pattern. Scale bar in all figures = 10 μm

We dissected brains of RN2-*Flp*, *Tub*-FRT-CD2-FRT-Gal4, UAS-*mCD8GFP *animals reared at 25°C and stained them with antibodies against green fluorescence protein (GFP; Figure 1a). 'Flippase' activity regulated by the RN2 promoter induces Flip Recombinase Target (FRT)-mediated recombination, leading to GFP expression in a small number of neurons in the adult central nervous system under *Tub*-Gal4 control (Additional file 1). In 20% (77/390) of preparations, a large field interneuron was labeled; in about half of these preparations, only one of the bilateral neurons was marked, allowing a clear visualization of its projection pattern (Figure 1a, h). The morphology as well as serotonin-immunoreactivity (Figure 2a–d) led us to identify the cell as the CSD interneuron (CSDn) described previously [15]. In larvae, the architecture of the CSDn is strikingly less complex than that of the adult, although the pattern shares several similarities (Figure 1b, i). At both developmental stages, the soma lies within a lateral cluster of cells and neurites ramify into the ipsilateral antennal lobe (short arrows in Figure 1a, b). The axon trunk traverses the brain towards the higher order neuropile and crosses the midline to terminate with profuse arborizations within the contralateral antennal lobe.

In approximately 60% of adult preparations, a few terminal branches cross the midline to innervate the antennal lobe ipsilateral to the soma (arrow in Figure 1c, h); these collaterals are not observed at the larval stage (Figure 1b, i). The larval olfactory system lacks an antennal commissure (Subhashini Srinivasan, unpublished observations), which in the adult carries contralateral branches of a majority of ORNs and some PNs that connect both lobes [4]. The terminals of the CSDn in the lobe contralateral to the soma are blebby in appearance, strongly serotonin positive (Figure 2b) and resemble structures of the *B. mori *CSD neuron that were shown by electron microscopy to be presynaptic to some of the neural elements within the lobe [20]. In order to confirm that these endings are presynaptic, we expressed a GFP-tagged synaptotagmin (Syt-GFP) in the CSDn and found the protein highly enriched in the terminal endings (Additional file 2c, f) and not in the ipsilateral arbors (Additional file 2a). The dendritic identity of the branches within the ipsilateral antennal lobe was ascertained using a strain expressing a microtubule motor Nod tagged with β-galactosidase (Nod::LacZ) [23]. Nod-β-galactosidase was enriched in a short region of the neuron beyond the cell soma including short branches within the ipsilateral lobe (arrowhead in inset of Figure 1d). Labeling was largely absent from the axon. Immunoreactivity against the Discs-Large (Dlg) protein has recently been used as marker of dendrites in the *Drosophila *antennal lobe [24]. We expressed a transgene where Dlg was tagged with GFP (Dlg-GFP) [25] using the RN2-*Flp*, *Tub*-FRT-CD2-FRT-Gal4 strain. The tagged protein was preferentially located in the branches within the ipsilateral lobe (arrowheads in Additional file 2b, f) and was very reduced in the terminal arbors (Additional file 2d). These findings, together with the observation that the branches do not accumulate the presynaptic protein Syt-GFP (Additional file 2a), lead us to conclude that the dendritic branches within the ipsilateral lobe are postsynaptic while those terminating in the contralateral lobe are presynaptic.

Branches (asterisks in Figure 1a, e) exit the trunk of the CSDn and traverse anterior to the mushroom body, to terminate in the posterior superior protocerebrum and the dorsal horn (arrowheads in Figure 1a). A few neurites from branches exiting the trunk in the hemisphere contralateral to the cell body terminate among the Kenyon cells in the calyx of the mushroom body (white arrows in Figure 1g). Only a subset of the arbors at the higher centers are stained with anti-serotonin antibodies (arrows in Figure 2c, d), and express Syt-GFP (Additional file 2c, d), raising the possibility that this region of the neuron has both pre-synaptic as well as post-synaptic endings. The putative presynaptic terminals in the ipsilateral higher centers are low in number; however, there are profuse serotonin-positive terminals from other neurons in the vicinity (red in Figure 2c). Electron microscopic analysis of the equivalent neurites in the moth showed spine-like projections, supporting the idea that some of the arbors of the CSDn in lateral protocerebrum are postsynaptic to other neurons in the region. [20]. Branching at the larval stages (Figure 1b, i) is much less profuse and arborization at the higher centers is restricted largely to the ipsilateral side with only a few small projections (yellow arrow in Figure 1b) on the contralateral side. The branch extending to the region of the calyx of the mushroom body is absent in the larva.

The CSDn is born during embryogenesis and is comparable in architecture during first, second and third instar stages (not shown). A conclusion that the neuron is related to a lineage specified by *even-skipped *(*eve*) is supported by the observation that other P (Gal4) lines regulated by *eve*-RRK-Gal4 and RN2-Gal4- [26], also drive GFP reporters in CSD neurons (data not shown).

### The dendritic and axonal arbors of the larval CSDn are sculpted during pupation to create a more complex adult architecture

**Figure 3:**
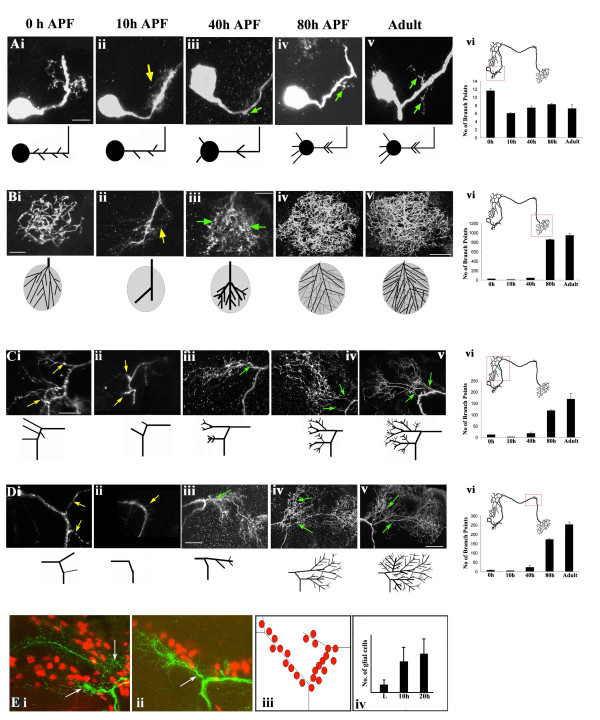
Re-modeling of the CSD neuron during pupal life. **(a-d) **Developmental changes in different regions of the CSD neuron; (a) cell body and dendrites within the ipsilateral lobe, (b) presynaptic terminals in the contralateral lobe, (c) branches in the ipsilateral higher centers, and (d) branches in the contralateral higher centers. The regions are shown at (i) 0 hours APF, (ii) 10 hours APF, (iii) 40 hours APF, (iv) 80 hours APF and (v) adult. Scale bar = 5 μm in (i) and (ii); 10 μm in (iii); 40 μm in (iv) and (v). Yellow arrows indicate branches being pruned while green arrows point to the new sprouts. The branching has been quantified as described in Additional file 3 and plotted in histograms in (a(vi)), (b(vi)), (c(vi)) and (d(vi)). The number of branch points is represented at different time points; bars indicate the mean and standard error of mean of five preparations in each case. **(e) **Infiltration of glial cells at the sites of pruning of the CSD neuron at (i) 10 hours and (ii) 20 hours APF. The glial cells are visualized by staining with an antibody against Repo (red). The means and standard error of mean of the number of cells in five preparations are represented in the histogram in (e(iv)).

The CSDn in the larva metamorphoses to a more complex adult pattern during pupal life. (Figure 3). The changes occurring at different positions of the cell were visualized in animals reared at 25°C and the branch point number estimated as described in Additional file 3. At the onset of pupariation (0 hours after puparium formation (APF)), CSDn morphology is identical to that in the third instar larva, but begins to undergo dramatic changes within the first few hours. Arbors from all regions of the neuron develop constrictions and blebs of GFP and severed branches become obvious by 10 hours APF (yellow arrows part (ii) of Figure 3a–d). Staining with an antibody against the reverse polarity protein (Repo) revealed the presence of a large number of glial cells in the vicinity of CSDn (Figure 3e(i)). The numbers increase until 20 hours APF and line the degenerating arbors (Figure 3e(ii–iv)). Glial cells are not observed in this pattern at 0 hours APF, consistent with the idea that these cells move into regions where pruning of neurons is taking place [27,28].

The earliest adult-specific outgrowth can be detected at 30 hours APF (not shown) and by 40 hours; all regions of CSDn begin to elaborate fresh arbors (green arrows in Figure 3a(iv), b(iii), c(iii, iv), d(iii, iv)), which continues until the final pattern is achieved by 80 hours APF (Figure 3b(iv, vi), c(iv, vi), d(iv, vi)). The change in neuronal morphology is particularly obvious in the terminals in the contralateral olfactory lobe (compare Figure 3b(iii) with 3b(ii)). The elaboration of the 'footprint' of the presynaptic terminals of CSDn accompanies the increase in volume of the developing antennal lobe. Modification of neuronal architecture involves an initial pruning of larval arbors that is complete within the first 20 hours of pupation followed by 'elaboration' of adult-specific branches. In subsequent descriptions we will refer to the early events (0–20 hours APF) as the 'pruning' phase and the 'later' events (30 hours to adulthood) as 'elaboration'.

The CSDn expresses serotonin during the larval stage and immunoreactivity is present through pupal life (data not shown). In order to test whether the changes in the pattern were regulated by levels of serotonin, we crossed a temperature sensitive allele of *dopa decarboxylase *(ddc^ts2^) into the RN2-*Flp*, *Tub*-FRT-CD2-FRT-Gal4, UAS-*mCD8GFP *strain. Dopa decarboxylase catalyses the biosynthesis of dopamine from tyrosine and serotonin from tryptophan. Growth of ddc^ts2 ^homozygous animals at non-permissive temperature (29°C) between the third larval instar stage and adulthood results in a striking decrease in serotonin immunoreactivity in the antennal lobe, although trace amounts still remained (Figure 2e). In these animals, the terminal branches of the CSDn and the general patterning of the neuron was unaffected within the resolution of our experiments; Figure 2f–h). This suggests that serotonin (or Dopa) does not regulate the metamorphosis of the CSD neuron during pupal life, at least at a gross level.

### Remodeling of CSDn is mediated through ecdysterone receptor signaling

**Figure 4:**
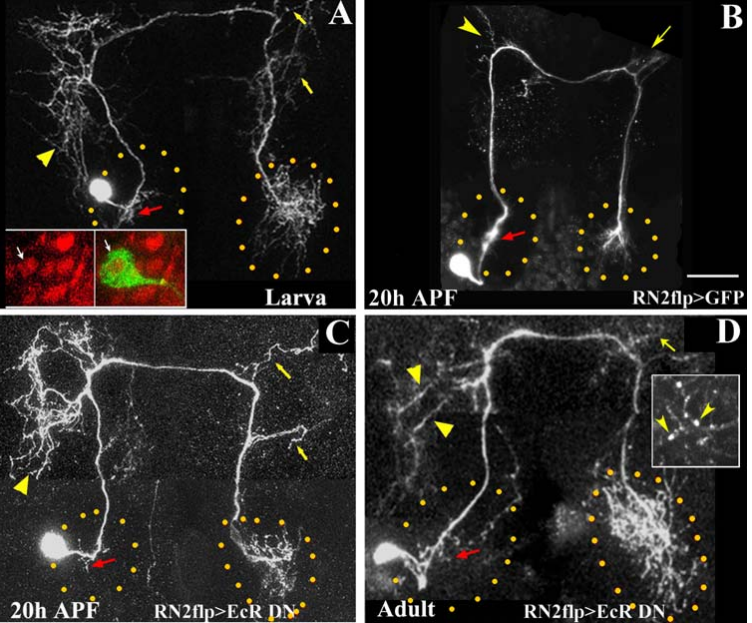
Role of ecdysterone signaling in remodeling of the CSD neuron. **(a, c, d) **CSDn profiles visualized in RN2-*Flp*, *Tub*-FRT-CD2-FRT-Gal4, UAS-*mCD8GFP*/UAS-*EcR*^W650A ^animals at different developmental stages. **(b) **RN2-*Flp*, *Tub*-FRT-CD2-FRT-Gal4, UAS-*mCD8GFP*/+ control at 20 hours APF; scale bar for all images = 30 μm. (a) In the larvae, branching within the antennal lobe (red arrow), the ipsilateral higher centers (arrowhead) and the terminals are comparable to that of the wild type (Figure 1b). Branches at the contralateral higher centers (yellow arrows) appear more elaborate than that of normal controls (Figure 1b). Staining with antibodies against the EcR-B1 isoform (red in inset) and anti-GFP (green in inset) demonstrated that the CSDn soma (arrows in inset) expressed EcR-B1. At 20 hours APF, pruning has occurred in the normal CSDn (b) and branching within the antennal lobes (demarcated with dotted lines), the ipsilateral (arrowheads) and contralateral (arrows) are greatly reduced. CSDn expressing a DN-EcR transgene (c) does not undergo pruning and the neuronal architecture resembles that of the larva. In the adult (d), the pattern continues to be 'larva-like'. However, the footprint of the terminal arbors occupies a larger area than in the larva (demarcated with yellow dots) and many of the arbors appear blebby (arrowheads in inset in (d)).

Steroid hormones in *Drosophila*, notably ecdysterone and its metabolite 20-hydroxyecdysterone, are known to regulate the rewiring of the adult central nervous system [29]. At the onset of pupation, the CSDn cell body shows immunoreactivity against common-epitope (not shown) and ecdysterone receptor B1 (EcR-B1; white arrows in inset in Figure 4a) but not EcR-A antibodies (not shown). The presence of the EcR-B1 isoform is indicative of neurons that undergo pruning during metamorphosis [30]. In order to block receptor function, we crossed UAS-*EcR-B1*^W650A ^into the RN2-*Flp, Tub*-FRT-CD2-Gal4, UAS-*mCD8-GFP *stock and reared the flies at 25°C. This dominant-negative form of the receptor (EcR-DN) is ligand-insensitive but is able to bind DNA and the EcR co-factor, Ultraspiracle (USP), thus competitively inhibiting the function of the wild-type receptor [31].

Targeted expression of EcR-DN abrogates metamorphosis of the CSDn, resulting in a 'larva-like' morphology in the adult brain (compare Figure 4d with Figure 1a). The architecture of the CSDn in the RN2-*Flp*, *Tub*-FRT-CD2-FRT-Gal4, UAS-*mCD8GFP*/UAS-*EcR*^W650A ^strain could be followed by co-expression of the GFP reporter. At the larval stage, the CSDn expressing EcR-DN is largely comparable to that of wild-type controls (compare with Figure 1b); we did, however, detect a few additional branches, emanating from the main trunk in the hemisphere contralateral to the soma (yellow arrows in Figure 4a). This phenotype suggests that proper signaling through EcR is necessary to maintain the pattern of the larval neuron; the GFP reporter indicated that expression is initiated only in the late embryo after the CSDn is patterned. At 20 hours APF, wild-type neurons show considerable pruning of neurites at the higher centers (yellow arrowhead and arrow in Figure 4b, the ipsilateral dendrites (red arrow in Figure 4b) and the terminals within the contralateral antennal lobe. None of these pruning events occur when CSDn expresses EcR-DN (Figure 4c) and the neuron continues to resemble that of the larva. This 'larva-like' pattern persists into adulthood (Figure 4d), although a few branches appear blebby and could be undergoing degeneration (inset in Figure 4d). The footprint of the terminal arbors within the contralateral antennal lobe (demarcated with dotted line) appears larger than that at the larval stage, suggesting that some re-growth has occurred. It is not clear whether the failure of elaboration of adult-specific branches indicates a requirement for EcR in this process or suggests that re-growth can occur only after pruning is completed.

### Ectopic expression of tetanus toxin, dominant negative *shibire *ansgene or modified inward rectifying K^+ ^channel affect the patterning of the adult CSDn

In order to test the effect of neural activity upon the proper development of the CSDn, we used several transgenes that act in different ways to silence neurons. First, targeted expression of the light chain of tetanus toxin (TeTxLC) has been shown to enzymatically cleave neuronal synaptobrevin, abolishing evoked synaptic release totally and diminishing spontaneous release by 50–75% [32,33]. Second, the *shibire *(*shi*) gene product Dynamin is essential for synaptic vesicle recycling, and ectopic expression of the semidominant *shi*^ts1 ^allele blocks neurotransmitter release [34]. Third, expression of an inwardly rectifying K^+ ^channel (Kir_2.1_) hyperpolarizes neurons, thereby inhibiting action potential generation [35].

**Figure 5:**
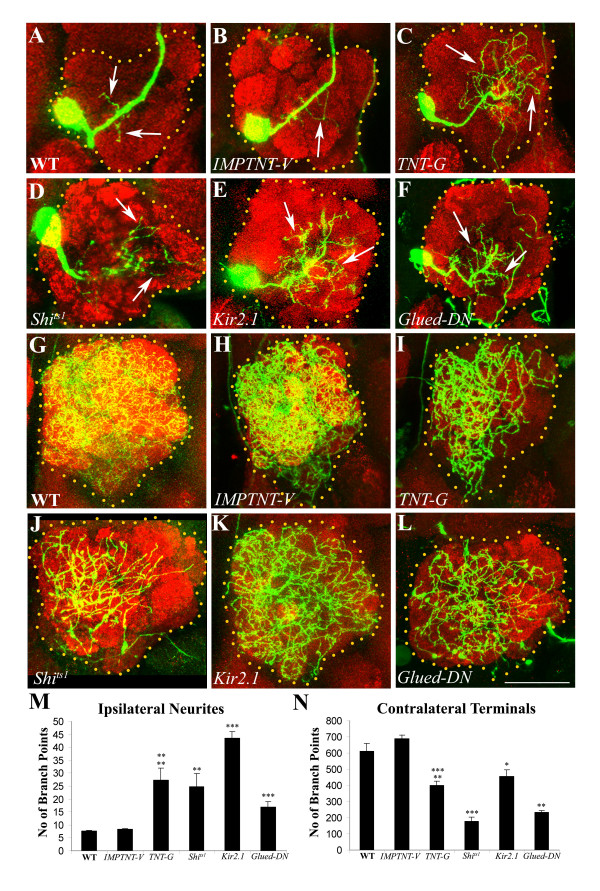
Targeted expression of TeTxLC, Shi^ts1^, Kir_2.1 _and Glued-DN affects the architecture of the CSDn. Adult brains were stained with anti-GFP (green) and mAbnc82 (red). **(a, g) **Wild-type (WT) CSDn showing the cell body with dendrites in the ipsilateral lobe (arrows in (a)) and terminal arbors in the contralateral lobe (g). **(b, h) **The neuron expressing inactive tetanus toxin (UAS-*IMPTNT-V*/+; RN2-*Flp, Tub*-FRT-CD2-FRT-Gal4, UAS-*CD8GFP*/+) have dendrites (arrow in (b)) and terminal arbors (h) comparable to WT. **(c, i) **Ectopic expression of active form of tetanus toxin (UAS-*TNT-G*/+; RN2-*Flp, Tub*-FRT-CD2-FRT-Gal4, UAS-*CD8GFP*/+) results in dramatic increase in ipsilateral dendrites (arrows in (c)) and a marked decrease in contralateral terminals (i). **(d, j) **UAS-*shi*^ts1^/+; RN2-*Flp, Tub*-FRT-CD2-FRT-Gal4, UAS-*CD8GFP*/UAS-*shi*^ts1 ^showing greater profusion of dendrites in the ipsilateral lobe (arrows in (d)) and reduced terminal arbors (j). **(e, k) **RN2-*Flp, TubFRT*-CD2-FRT-Gal4, UAS-*CD8GFP*/UAS-*EGFPKir*_2.1 _showing greatly increased dendritic branches within the ipsilateral lobe (arrows in (e)) and modest decrease in contralateral terminals (k). **(f, l) **UAS-*Glued-DN*/+; RN2-*Flp, Tub*-FRT-CD2-FRT-Gal4, UAS-*CD8GFP*/+ showing increased ipsilateral dendrites (arrows in (f)) and a reduction in contralateral terminals (l). Scale bar = 30 μm. **(m, n) **Number of branch points from the ipsilateral (m) (N = 7) and contralateral (n) (N = 5) arbors were quantified from all the genotypes. Bars represent the mean and standard error of mean of number of branch points. *P*-values were calculated using the unpaired Student's *t*-test *P < 0.05, **P < 0.01, ***P < 0.0001.

We used the RN2-*Flp*, *Tub*-FRT-CD2-FRT-Gal4, UAS-*mCD8-GFP *stock to drive expression of each of these activity blockers in the CSDn neuron and compared the pattern of branching in the lobe ipsilateral and contralateral to the cell soma with that of the wild type (Figure 5). Since the neuron also expresses GFP, we could examine its morphology in detail and also quantify the branching pattern as described above (Additional file 3; Figure 5m, n). In order to analyze effects on the ipsilateral arbors of CSDn we selected those preparations (approximately 40%) that lacked projections from the contralateral terminals that cross the midline, as these hinder clear visualization of the ipsilateral branches (Figure 1c, h). We independently ascertained that the defects observed upon perturbation were not due to the lack of the contralateral projection (not shown). The normal pattern of the CSDn (Figure 5a, g) was only slightly altered by targeted expression of a mutated and inactivated form of tetanus toxin (IMPTNT-V; Figure 5b, h, n). We do find evidence for some residual activity of in the IMPTNT-V transgene in several of our studies (unpublished data). However, dramatic changes were seen when the activated form of tetanus toxin (TNT-G; Figure 5c, i) was expressed. The number of ipsilateral branches was greatly increased (*P *< 0.001; Figure 5c, m) while the terminal endings in the contralateral lobe were reduced (*P *< 0.001; Figure 5i, n;). Similar effects were observed in the adult dendritic pattern upon transgenic expression of Shi^ts-1 ^(Figure 5d, m) or Kir_2.1_(Figure 5e, m). In both cases the CSDn produced profuse dendrites invading the ipsilateral lobe (*P *= 0.001 for Shi^ts-1^; *P *= 0.0001 for Kir_2.1_; compare Figures 5d, e and 5a). As described for TNT-G expression, the extent of branching of presynaptic terminals in the contralateral lobe was strikingly reduced in UAS-*shi*^ts-1^/+; RN2-*Flp*, *Tub*-FRT-CD2-FRT-Gal4, UAS-*mCD8-GFP*/UAS-*shi*^ts-1 ^animals (*P *= 0.0001; Figure 5j). The effect on terminal branching by Kir_2.1_expression was also seen (*P *= 0.03; Figure 5k), although this was less striking than that with TNT-G or *shi*^ts-1 ^expression.

Our data show that blocking synaptic release by TeTxLC or by a Dynamin mutation alters the architecture of both the dendritic as well as presynaptic arbors of the CSDn. The decrease in branching in the contralateral antennal lobe could, in principle, be explained by the effects of TNT-G and dominant negative Shibire on vesicle recycling and, hence, axon growth cone dynamics. However, the increased dendritic arborization within the ipsilateral lobe is more likely to be due to blocking of neuronal activity. This effect is very pronounced in animals where the neuron is silenced by expression of Kir_2.1_, which acts by hyperpolarizing the membrane and, hence, abolishing both spontaneous and evoked activity.

### Targeted expression of truncated Glued affects CSDn dendritic arbors and terminal branches

Several lines of evidence suggest that retrograde transport plays an essential role during pathfinding and synapse formation [36] and may form a link between activity and cell growth (reviewed in [37]). A truncated form of Glued (Glued-DN), when expressed under the UAS promoter, disrupts Dynein-dependant retrograde transport [36]. We tested the effect of the expression of this construct during CSDn metamorphosis. Interestingly, the effect of Glued-DN expression on patterning was similar to that seen upon silencing of activity within CSDn (Figure 5f). The ipsilateral dendrites were greatly increased in number (*P *< 0.0001; Figure 5f, m;) and the presynaptic terminals were reduced (*P *< 0.0003; Figure 5l, n;). The effects on the presysnaptic terminals are consistent with a requirement for retrograde transport during synaptic terminal formation. The increased ipsilateral dendrites could be a secondary consequence of altered synapse formation at the terminals or because retrograde signals that regulate formation of postsynaptic structures are rendered defective by the expression of the Glued-DN protein.

### Expression of tetanus toxin affects elaboration of new branches but not pruning events during CSDn metamorphosis

**Figure 6:**
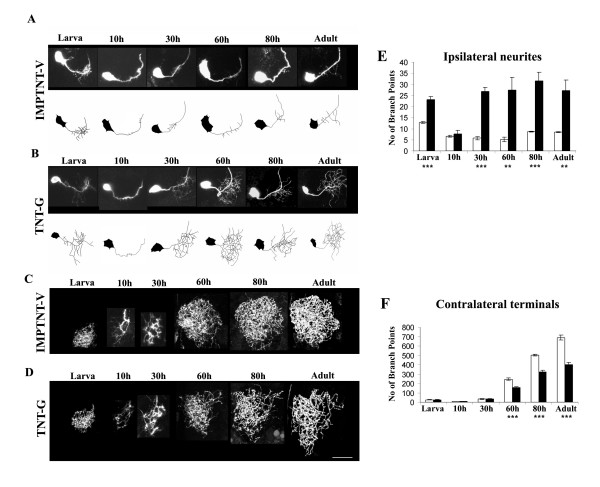
Effect of expression of TeTxLC on the remodeling of the CSD neuron. The developmental profile of the CSDn in animals where the **(a, c) **inactivated (UAS-*IMPTNT-V*/+; RN2-*Flp, Tub*-FRT-CD2-FRT-Gal4, UAS-*CD8GFP*/+) and **(b, d) **activated (UAS-*TNT-G*/+; RN2-*Flp, Tub*-FRT-CD2-FRT-Gal4, UAS-*CD8GFP*/+) forms of TeTxLC have been expressed. Animals where a unilateral flipout had occurred were analyzed in detail at the larva, 10 hour APF, 30 hour APF, 60 hour APF, 80 hour APF and adult stages. Scale bar for all images = 30 μm. The dendritic trees from preparations where inactivated (a) and activated (b) TeTxLC have been ectopically expressed are schematized. The presynaptic terminals in the contralateral lobe where IMPTNT-V and TNT-G were expressed are shown in (c) and (d), respectively. The branch points were quantified as described in Additional file 3 and represented in the histograms in **(e) **for the ipsilateral lobe and **(f) **for the contralateral lobe. Five preparations were analyzed at each data point; white bars represent UAS-*IMPTNT-V*/+; RN2-*Flp, Tub*-FRT-CD2-FRT-Gal4, UAS-*CD8GFP*/+; black bars represent UAS-*TNTG*/+; RN2-*Flp, Tub*FRT-CD2-FRTGal4, UAS-*CD8GFP*/+. *P*-values were calculated using the unpaired Student's *t*-test; **P < 0.001, ***P < 0.0001.

As described above we targeted expression of an inactivated (IMPTNT-V; Figure 6a, c) or activated (Figure 6b, d) form of tetanus toxin into the CSDn and examined the metamorphosis of the CSDn by visualizing GFP expression. Neurons expressing the inactive toxin (Figure 6a, c) showed normal kinetics of pruning and elaboration. TNT-G affects the metamorphosis of the CSDn with different effects on the ipsilateral dendrites (Figure 6b, e) and the presynaptic terminals in the contralateral lobe (Figure 6d, f). At the larval stage there are an elevated number of dendritic branches in the ipsilateral lobe (Figure 6b) compared to CSDn expressing IMPTNT-V (*P *= 0.0007; Figure 6a). These 'excess' arbors are removed by pruning in the 10 hour APF animal (Figure 4b), making it comparable to control genotypes (*P *= 0.96; Figure 6a). A phase of re-growth begins by 30 hours APF in both IMPTNT-V (Figure 6a) and TNT-G (Figure 6b) expressing neurons, although the number of branches is more than three-fold higher in the latter (*P *= 0.0001). These 'ectopic' dendrites remain until adulthood (Figure 6b).

In addition to a profusion of dendritic arbors, TNT-G-expressing adult neurons show a reduction in branching of terminal arbors (*P *= 0.003; Figure 6d). The defect occurs during development of adult-specific arbors since the larval terminals and pruning are normal (*P *= 0.37; compare Figures 6c and 6d, f). These studies show that blocking neuronal synaptobrevin has different effects on the dendrites and presynaptic terminals of the CSDn. The presynaptic terminals show reduced elaboration, which is consistent with an effect of TNT-G on vesicle recycling and, hence, axon growth. The dendrites, on the other hand, show excess growth and branching, a phenotype that is more likely to be a result of a lack of activity in the neuron rather than growth.

### ORNs or mushroom bodies are not essential for CSDn architecture while the presence of lobe interneurons in the antennal lobe influences the branching pattern of the neuron terminals

**Figure 7:**
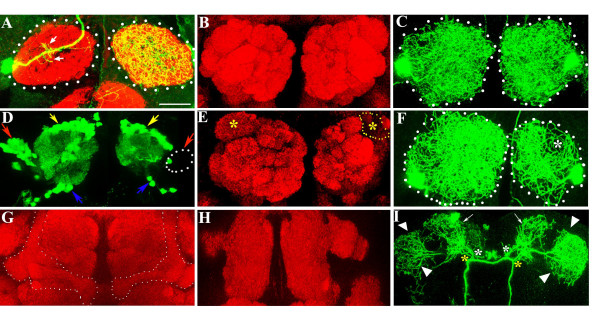
Effect of ablation of subsets of neurons within the olfactory circuit on the development of the CSD neuron. **(a) **Antennal lobes (demarcated with dotted lines) of *lz*^3^; +/+; *Antp*/RN2-*Flp, Tub*-FRT-CD2-FRT-Gal4, UAS-*CD8GFP *stained with anti-GFP (green) and mAbnc82 (red). *lz*^3^; +/+; *Antp*/+ animals lack ORNs projecting from the third antennal segment. As in the wild-type (Figure 1a), the CSDn in this genetic background sends a few dendrites into the ipsilateral lobe (small arrows) and terminates by extensive branching in the lobe contralateral to the cell body. The glomeruli are less well demarcated by mAbnc82 staining compared to control brains shown in **(b)**. **(c) **RN2-*Flp, Tub*-FRT-CD2-FRT-Gal4, UAS-*CD8GFP *where terminals of both CSDn are labeled within the antennal lobes. **(d-f, h, i) **Brains of animals that had been fed with HU, stained with anti-GFP (green) and mAbnc82 (red). (d, e) *GH146*-Gal4, UAS-*GFP*. (d) Cell bodies of the PNs lie in three clusters located anterodorsal (blue arrows), posterior (yellow arrows) and lateral (red arrows) to the antennal lobe. A subset of the lateral cluster neurons (dotted region) is absent in one lobe. (e) This results in the absence of a glomerulus denoted with (*-DA1) on the affected side (yellow dotted lines). (f) RN2-*Flp, Tub*-FRT-CD2-FRT-Gal4, UAS-*mCD8GFP *animals treated as the *GH146*-Gal4, UAS-*GFP *animals described above. Animals with one lobe reduced in size were selected and the terminal arbors from bilateral flip-outs examined. The affected side shows significantly less branching, particularly over the region where the DA1 is positioned (asterisk; compare with (c)). **(g) **Mushroom bodies are indicated by dotted lines in control (no HU) preparations. (h, i) In HU treated animals where the mushroom bodies were absent (h), the architecture of the CSDn was normal (i). Secondary branches (yellow and white asterisk) that arborize over the calyx of the mushroom bodies (arrows) and the lateral horn (arrowheads) appear normal. Arrows indicate branching at the calyx region and arrowheads the branching at the lateral horn. Scale bar = 20 μm.

We asked whether neurons within the olfactory circuit directly or indirectly regulate the morphology of the CSDn. At the larval stage, approximately 21 ORNs project from the olfactory organ to the antennal lobe [38]; these neurons begin to degenerate at the onset of pupation. Adult ORNs develop from the eye-antennal imaginal disc and project to the developing adult lobe by 18 hours APF [39,40]. The axons circumscribe the surface of the lobe by 20 hours APF and begin to invade the neuropile by approximately 25 hours APF. Glomerular formation at the macroscopic level is completed by about 60 hours APF. In order to test whether the newly forming ORNs provide cues for CSDn development, we generated flies in which the third antennal segment was completely converted to a leg, thus leading to absence of ORNs throughout development. Transformation of the antenna to the leg is achieved by the dominant mutation *Antennapedia *(*Antp*); a few trichoid sensilla are still present and these can be abolished using strongalleles of *lozenge *(*lz*^3^). When *lz*^3^;*Antp/+ *animals are stained with the synaptic marker mAbnc82 [41], the olfactory glomeruli are poorly defined (compare Figures 7a and 7b). The architecture of the CSDn however, closely resembles that of the wild type (compare Figures 7a and 1a), suggesting that the neuronal remodeling is not guided by sensory input from the antenna. It must be mentioned that ORNs from the maxillary palp that project to the antennal lobe are unaffected in *Antp *mutants.

PNs and LNs are present within the antennal lobe anlagen before the arrival of the adult ORNs [39]. Clonal analysis, as well as neuroblast ablation by feeding larvae with the ribonucleotide reductase inhibitor hydroxyurea (HU), has mapped the lineage of interneurons within the antennal lobe [42,43]. Cell bodies of the approximately 90 PNs marked by expression of GFP in the *GH146*-Gal4 lie in three clusters: anterodorsal (approximately 50; yellow arrows in Figure 7d), lateral (approximately 35; red arrows in Figure 7d) and ventral (approximately 6; blue arrows in Figure 7d) to the antennal lobe. Clonal analysis demonstrated that each PN cluster arises from a single neuroblast during the embryonic and larval stages [42]. Feeding newly hatched larvae with HU for four hours results in ablation of a large subset of lateral cluster neurons (Figure 7d, dotted lines) and a resultant effect on specific glomeruli (Figure 7e, yellow dotted region). Several LNs are also ablated by HU feeding at this time in larval life, but the majority of these cells have multi-glomerular targets and the effects are likely to be more widespread through the lobe (Abhijit Das, personal communication). The absence of PNs arising from the lateral neuroblast leads to a reduction in size of target glomeruli, for example, DA1 (marked with an asterisk in Figure 7). The presynaptic terminals of CSDn in these reduced antennal lobes showed a smaller 'footprint' (Figure 7f, dotted lines) as well as reduced branching (asterisk in Figure 7f). The change in branching pattern was particularly noticeable in the vicinity of the affected glomeruli. We speculate that proper patterning of the CSDn requires its synaptic partners, which are likely to be the dendrites of the PNs and possibly also the LNs. This connectivity needs to be confirmed by electron microscopic analysis.

In addition to the PN/LN progenitors, HU treatment during this period also affects neuroblasts contributing to the mushroom body neuropile [44]. In animals in which the mushroom bodies (Figure 7g) are absent or greatly reduced in size with no change in the PNs (N = 5; Figure 7h), branches of CSDn in the region of mushroom bodies (Figure 7i, arrows), lateral protocerebrum (Figure 7i, arrowheads) as well as the antennal lobes (not shown) are unaffected. This is not surprising since while the CSDn branches extensively (white asterisk in Figure 7i), only a few branches actually innervate the calyx of the mushroom bodies (Figure 1).

## Discussion

Changes in the pattern of arborization of a mature neuron can come about as a consequence of removal of its afferent inputs or targets, chronic stress or other environmental inputs, such as delivered during learning or exercise [45]. Many of these changes are effected through the action of growth factors and developmental signals acting in concert with steroid hormones and neuronal activity to modify the cytoskeleton or synaptic properties relevant to an altered functional setting. Metamorphosis in *Drosophila *– a period during which mature larval neurons are often altered to take on new adult functions – provides a context where the mechanistic underpinnings of such neuronal change can be genetically dissected.

In this study we use a genetic method to mark the CSDn, recently identified on the basis of serotonin immunoreactivity [15]. While this preparation identifies a central neuron, it also has an important feature that allows the analysis of mechanisms underlying the changes it undergoes during remodeling. Our system, because of the random nature of the RN2-FLP action, results in bilateral, unilateral or no excision of the FRT element in the Tub-FRT-CD2-FRT-Gal4 construct in the CSDn. Thus, we are able to choose and analyze preparations where the CSDn from only one hemisphere is labeled: this facility is vital as it allows the analysis of contralateral and ipsilateral projections of the CSDn, without this being obscured by projections of the neuron from the other hemisphere to the same target sites. The GFP reporter in the RN2-*Flp*, *Tub*-FRT-CD2-FRT-Gal4, UAS *mCD8-GFP *strain is first detected very late in embryogenesis (stage 20; data not shown), after the neuron has acquired its mature larval pattern. These features thus provide a preparation where an identified central neuron, whose function is known, can be followed and genetically manipulated as it changes its form in response to external and internal cues during metamorphosis.

We show that the neuron, present during the larval stages, undergoes well-defined changes during pupation to give rise to a more complex adult architecture. What are the factors that regulate the stereotyped pruning and re-growth of arbors in the CSDn during metamorphosis? Our results suggest that the interaction of external factors and autonomous properties – some of which we identify – establish the homeostasis required during branching and establishment of the adult form.

### Metamorphosis of the CSD neuron provides a useful model for the study of events during pruning

Arbors from the larval neuron are removed by pruning over the first 20 hours of pupation before the adult pattern is elaborated. TheEcR-B1, isoform whose expression is typically seen in neurons that alter their larval form and contribute to the circuitry in the adult [46], is detected in CSDn. Down-regulating EcR in the CSDn during metamorphosis results in a failure of remodeling and the 'adult' neuron retains a larval morphology. The detailed mechanisms by which EcR signaling acts to bring about sculpting of cell shape are not totally understood and reports on *Manduca sexta *indicate that steroid-induced modifications in dendritic shape can be regulated by activity-dependent mechanisms [47].

Studies on the cellular and molecular mechanisms of pruning events during metamorphosis could provide valuable insights into our understanding of degeneration in higher systems. These events require ubiquitin-mediated proteolysis [28] and it is known that local activity of caspases is involved in dendritic pruning in an identified sensory neuron [48]. Degeneration of specific branches is followed by migration of glial cells into the site of activity [27,28]. The role of these glia in bringing about pruning and in clearing debris from the vicinity requires further study.

### Does neural activity play a role in shaping the arbors of the CSDn during pupation?

The assembly of complex circuits is dependent on a carefully orchestrated interplay of intrinsic and extrinsic cues [49]. Does activity play a role in determining neuronal shape? We silenced spontaneous and evoked activity in the CSDn using different methods and have observed changes in the dendritic arbors as well as in presynaptic terminals. The effects on the terminals and dendrites are possibly due to distinct mechanisms and will be discussed separately.

The strongest effects on presynaptic terminal branching were produced by expression of TeTxLC, which blocks synaptic release, and a dominant-negative Shi protein, which affects receptor-mediated endocytosis. Apart from blocking neuronal activity by abrogating synaptic vesicle release, both treatments could potentially affect axon growth. Consistent with this is the observation that TeTxLC expression affects re-growth of CSDn terminals during metamorphosis, while pruning occurred normally. Weak anatomical defects have also been described in other, non-modulatory neurons [50], some of which could be explained by a role in the regulation of levels of cell adhesion molecules [51].

Increases in size and branching pattern of the dendritic trees is a robust effect occurring notably when neuronal activity was silenced by Kir_2.1_expression (Figure 5). In the third instar larva, expression of TNT-G leads to an increase in dendritic arbors with no significant effect on the presynaptic terminals. Expression using the RN2-*Flp*, *Tub*-FRT-CD2-FRT-Gal4, stock initiates in the fully developed larval neuron; hence, the changes in dendritic branches are likely to be a consequence of lack of neuronal activity, rather than a developmental effect. What are the mechanisms by which neuronal activity can alter morphologies of neurons? Baines and co-workers [32] demonstrated that tetanus toxin expression in motorneurons not only affected its presynaptic release because of cleavage of synaptobrevin, but also altered synaptic input by an as yet unknown mechanism. Our finding of altered dendritic morphology supports the possibility that homeostatic alterations occur to compensate for a lack of activity.

A large body of data provides evidence for retrograde signaling in the development and consolidation of synapses [36,52] (reviewed in [53,54]). Our observation of expanded dendritic trees upon expression of a dominant negative form of Glued, while intriguing, is difficult to explain in this light. The changes we see are in the dendritic (post-synaptic) field when retrograde transport is blocked cell-autonomously. While this needs further investigation, a possible explanation is that these effects are an indirect consequence of physiological alterations at the presynaptic terminals. Local morphological changes in neurons can be effected by sequestration of proteosomes and other molecules at different regions of the cell in response to activity [55,56], which could result in sculpting of cellular architecture due to altered protein composition at different cellular regions.

Defects in branching observed by abrogation of vesicle release at the synapse in a serotonergic neuron could implicate this modulator in paracrine or autocrine signaling in regulation of neuronal outgrowth, target selection and synapse formation [57]. Such effects have been demonstrated in the gastropod *Helisoma *[58], as well as in *Drosophila*, where serotonin levels regulate neuronal branching [59] and modulate the development of neuronal varicosities in the central nervous system [60]. In our experiments, we failed to detect significant changes in the branching pattern of CSDn upon strong reduction of serotonin (and dopamine) using a temperature sensitive allele of *dopa decarboxylase*. Furthermore, unlike in *M. sexta*, where afferents are necessary for the formation of glomerular tufts of the serotonergic neuron within the antennal lobe [61], development of the CSDn occurs normally in the absence of sensory input from the antenna.

### Feedback neurons in the olfactory pathway

The olfactory pathway consists of afferent sensory neurons, local integrating neurons and projection neurons. Circuitry for an additional level of integration exists in the atypical projection neurons (aPNs), the antennal posterior superior protocerebral neuron (APSP), the giant symmetric relay interneurons (GSI) and the bilateral ACT relay interneurons (bACT) [12,13]. The architecture as well as the serotonergic nature of the CSDn closely resembles the S1 neuron in *M. sexta*, which receives input from bilateral projections in the protocerebrum and terminates in the lobe contralateral to the soma to modulate the activity of interneurons [17,21]. We propose that the ipsilateral dendrites receive input from as-yet unidentified neural elements in the antennal lobe, while some axonal arbors are postsynaptic to interneurons in the calyx of the mushroom bodies and the lateral horn. We speculate that the targets of the terminal arbors are either the PNs or the LNs since their ablation results in a reduction in branching. This architecture, which needs to be confirmed by electron microscopic analysis, provides circuitry for 'top-down' regulation of the primary olfactory center. It seems very likely that the CSDn, like its counterpart in the moth, responds to mechanosensory stimulation [20], providing an important role in responses to odor stimulation coupled with airflow, as would be expected in insects during flight. The modulatory effects of this large field neuron on its partners in the antennal lobe needs to be investigated by high-resolution functional imaging.

## Conclusion

We describe a serotonergic neuron whose anatomy suggests feedback integration within the antennal lobe of insects. The neuron undergoes remodeling during pupal life from a simple larval to a more complex adult pattern. Our studies suggest that the morphology of the dendritic arbors that terminate in the lobe ipsilateral to the soma is regulated by neuronal activity. The arborization of terminal arbors depends on vesicle recycling, endocytosis and Dynein-dependant retrograde transport. These findings demonstrate a useful identified-neuronal preparation where developmental mechanisms and remodeling can be studied in the context of olfactory behavior.

## Materials and methods

### Fly stocks

Flies of the genotype RN2-*Flp*, *Tub*-FRT-CD2-FRT-Gal4, UAS *mCD8-GFP *were used in our experiments to label the CSDn. The RN2-*Flp *transgene was constructed by fusing the regulatory region of the *evenskipped *(*eve*) locus necessary for expression in RP2, aCC and pCC neurons [26] to the Flippase coding region. The construct on the third chromosome was allowed to undergo recombination with a strain carrying the P-(*Tub*-FRT-CD2-FRT-Gal4) [62] cassette and also a UAS-*mCD8-GFP *transgene [63]. In flies of the RN2-Flp, *Tub*-FRT-CD2-FRT-Gal4, UAS-*mCD8-GFP *genotype, expression of Flippase in specific cells induces recombination at the FRT sites, hence inducing expression of GFP under *Tub*-Gal4 control. The UAS-*EcR-B1-d655W650A *stock (referred to as UAS-*EcR-B1*^W650A ^[31,64]) was obtained from the Bloomington *Drosophila *Stock center, Indiana. UAS-*TNT-G *and UAS -*IMPTNT-V *stocks were kindly provided by Sean Sweeney [33]. The IMPTNT-V is mutated in LC2V233 to V237 mutation, rendering the toxin essentially ineffective [33]. Expression of the inactive toxin was used as a control in all experiments. The UAS-*shi*^ts1^/UAS-*shi*^ts1^; +/+;UAS-*shi*^ts1^/UAS-*shi*^ts1^stock [34], expresses a temperature sensitive form of Dynamin, under GAL4 control, at 29°C, which results in a block in endocytosis. The construction of the *y, w*; UAS-*EGFP-Kir2.1 *transformant has been described in [35]. Targeted expression leads to an inward flux of K^+ ^ions that hyperpolarizes the resting membrane, resulting in silencing of neuronal activity. The *lz*^3 ^stock was obtained from R Stocker [65]. Mushroom body marking was achieved using the mb247-DsRed stock kindly provided by Andre Fiala. UAS-*nSybGFP *was a gift from Mani Ramaswami, UAS-nod:lacZ from Clark *et al*. [23] and UAS-Dlg from Ulrich Thomas [25]. The UAS-Syt-GFP stock, *Antennapaedia *(*Antp*^6^), RN2-Gal4 and the temperature sensitive allele *ddc*^ts2 ^were obtained from the Bloomington Stock Centre, Indiana.

All fly stocks were grown on standard cornmeal medium at 25°C. White prepupae (0 hours APF) were collected and allowed to develop on moist filter paper. RN2-*Flp*, *Tub*-FRT-CD2-FRT Gal4, UAS *mCD8-GFP *pupae take about 100 hours to eclose when grown in our laboratory. All perturbations were done at 25°C except for the experiments with *shi*^ts1 ^ectopic expression, for which animals were reared at 29°C.

### Immunohistochemistry

Dissection of pupal and adult brains and wholemount antibody staining was carried out as described in [40,66]. Dissections and fixation steps of the protocol were carried out on ice when serotonin immunohistochemistry was to be carried out. Primary antibodies used were mouse anti-Bruchpilot/mAbnc82 [41], rabbit anti-GFP (Molecular Probes, Invitrogen, Delhi, India; ; 1:10,000), mouse anti-Repo (Developmental Studies Hybridoma Bank. Univ. of Iowa, USA; 1:20), mouse anti-Ecdysterone receptors A, B1 and common-epitope (DSHB; 1:50), rabbit anti-serotonin (Sigma-Aldrich, Delhi, India; 1:5,000). Secondary antibodies used were Alexa 488 and Alexa-568 coupled goat anti-mouse and anti-rabbit IgG (Molecular Probes; 1:200). Samples were mounted between coverslips with a spacer in anti-fading agent, Vectashield (Vector Labs, Peterborough, U.K.,, imaged on Bio-Rad 1024 (United Kingdom) at 1 μm intervals; data were processed using Confocal Assistant 5.0, Image J 1.37a (Wayne Rasband, NIH, USA) and Adobe Photoshop 7.0 (Adobe Systems, San Jose, CA, USA).

### Image analysis

The number of branch points were estimated at the ipsilateral dendrites, presynaptic terminals in the contralateral lobe, the ipsilateral branches and the contralateral branches to the higher centers as described in Additional file 3. The neuron was imaged in 1 μm confocal sections through brain. Staining used antibodies against GFP. Each section was projected onto a monitor and branch points were marked with a dot on a cellulose acetate sheet. The total number of branches was counted throughout the z-stack; data were sampled from at least five preparations and mean and standard deviations were plotted in a histogram. Tests of significance were carried out using the Student's unpaired *t*-test.

To estimate the numbers of glial cells, Repo positive cells were counted in the near vicinity of the arbors of the CSD neuron over different developmental stages. The numbers of glia were estimated in five preparations and the mean and standard deviations plotted in a histogram.

### Neuronal ablation using hydroxyurea

HU containing yeast paste was prepared by mixing 25 mg into 500 μl of yeast paste solution [44]. Larvae of the genotype RN2-*Flp*, *Tub*-FRT-CD2-FRT Gal4, UAS *mCD8-GFP *were collected from the media within one hour of hatching and placed in either control or HU containing yeast paste. After four hours at 25°C, larvae were rinsed thoroughly in distilled water and placed in vials with standard fly food until adult eclosion. Adults were dissected and stained with anti-GFP to visualize the CSDn and mAbnc82 to visualize the antennal lobe. Confocal scans of control and experimental preparations were analyzed by an independent investigator who selected samples based on the reduced lobe size; the numbers of branch points of the CSDn terminals in these lobes were estimated.

## Competing interests

The author(s) declare that they have no competing interests.

## Authors' contributions

BR performed the developmental studies, analyzed the results and studied the effects of activity during metamorphosis of the CSD neuron. APS and CS carried out the ecdysteroid receptor ablations and hydroxyurea experiments while VC assisted in some of the analysis. AN and ML generated the flip-out strains. KVR, ML and VR conceived the experiments and participated in the preparation of the manuscript. All authors have read and approved the manuscript.

## Supplementary Material

Additional File 1 Broad expression pattern of RN2-*Flp, Tub*-FRT-CD2-FRT-Gal4, UAS-*mCD8GFP *in adult brain. **(a) **Large set of neurons marked as a result of flp activity in RN2-*Flp, Tub*-FRT-CD2-FRT-Gal4, UAS-*mCD8GFP *stock. **(b) **Both the CSD neurons (asterisk) have been marked (bilateral flipout). **(c, d) **A small set of central neurons (arrowheads). Cell body of the CSDn is indicated by an asterisk in (c). Scale bar = 30 μm. Click here for file

Additional File 2 Localization of Dlg-GFP and Syt-GFP in the larval CSD neuron. **(a, c) **RN2-*Flp, Tub*-FRT-CD2-FRT-Gal4/UAS-*Syt-GFP*. **(b, d) **UAS-*Dlg-GFP*/+; RN2-*Flp, Tub*-FRT-CD2-FRT-Gal4. (a, b) Ipsilateral dendrites; (c, d) contralateral presynaptic terminals stained with anti-GFP (green) and mAbnc82 (red). Syt-GFP localizes to the cell body but does not extend beyond a few microns in the primary neurite (asterisk; (a, a')), but is enriched within the synaptic endings in the antennal lobe (dotted lines) appearing as punctate staining (c, c'). Dlg-GFP is localized mainly within the cell body and ipsilateral neurites (arrowheads in (b, b') and is very weakly present in the terminal arbors within the antennal lobe (dotted lines in (d, d'). (e, e') Syt-GFP shows punctate localization in the branches at ipsilateral (arrow) and contralateral higher centers (arrowhead in (e, e'). (f) Diagrammatic representation of Syt-GFP (green) and Dlg-GFP (red) expression. Dlg-GFP is enriched in the dendritic trees while Syt-GFP is localized to the presynaptic terminals of CSDn. Click here for file

Additional File 3 Quantification of branch points. **(a-d) **Schematic of the branch point analysis of dendrites within the ipsilateral antennal lobe (a-c) and the contralateral terminals (d). (a, d) RN2-*Flp, Tub*-FRT-CD2-FRT-Gal4, UAS-*mCD8GFP/+ *dendritic (a) and terminal (d) arbors. (b) RN2-*Flp, Tub*-FRT-CD2-FRT-Gal4, UAS-*mCD8GFP/*UAS-*EGFPKir2.1*.(c) UAS-*shi*^ts1^/+; RN2-*Flp, Tub*-FRT-CD2-FRT-Gal4, UAS-*CD8GFP*/UAS-*shi*^ts1^. **(e) **Schematic showing that the neuron was imaged in 1 μm confocal sections through the brain. Each section was projected onto a monitor and the branch point denoted by a red dot on a cellulose acetate transparent sheet. The total number of branch points was counted over the whole z-stack. Click here for file
