# Supplementary figures and images for: Metamorphosis of an identified serotonergic neuron in the Drosophila olfactory system
Source: Neural Dev. 2007 Oct 24;2:20. doi: 10.1186/1749-8104-2-20 (PMC2129096; doi:10.1186/1749-8104-2-20)

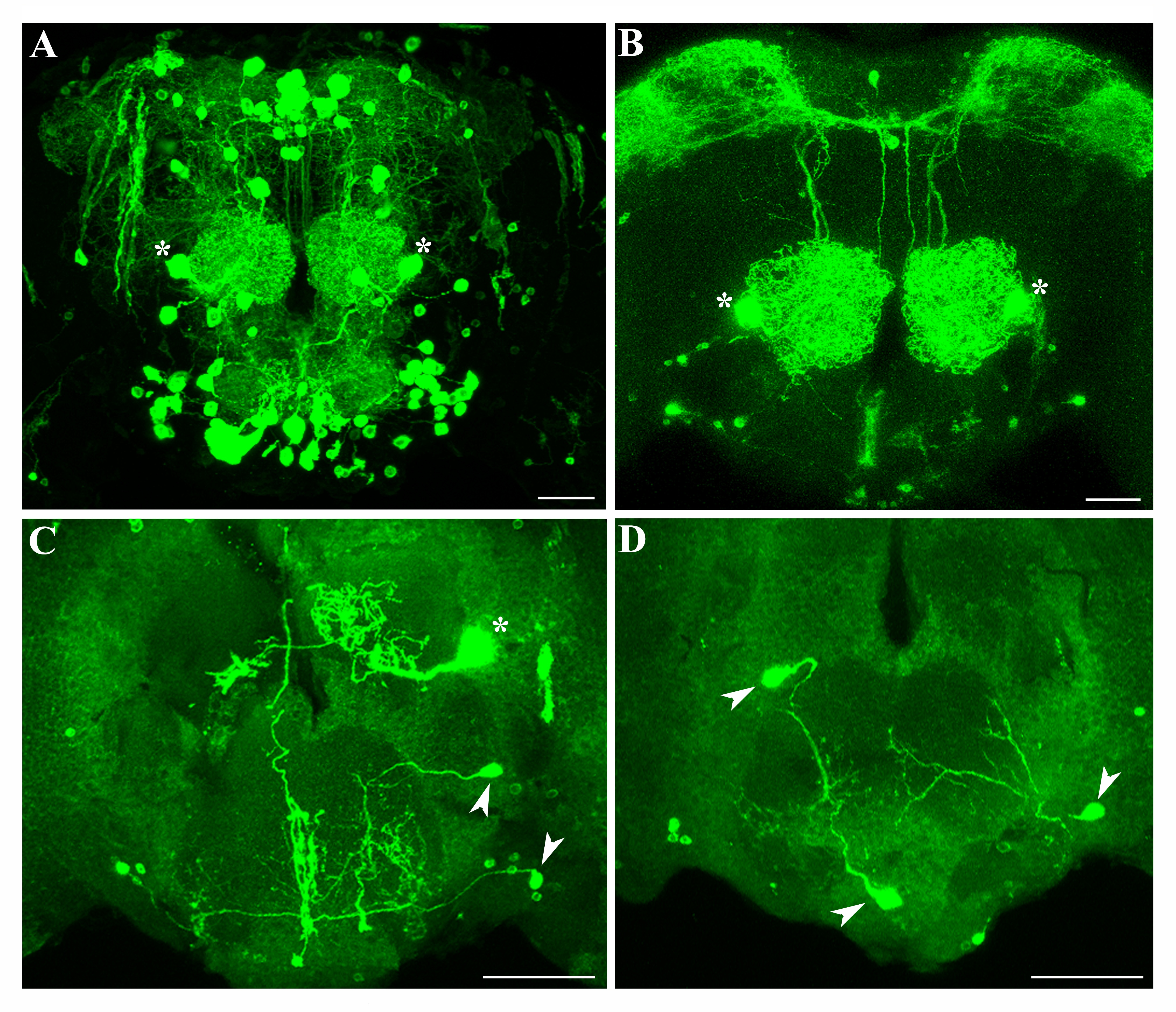

Supplement: Additional File 1 — Broad expression pattern of RN2-Flp, Tub-FRT-CD2-FRT-Gal4, UAS-mCD8GFP in adult brain. (a) Large set of neurons marked as a result of flp activity in RN2-Flp, Tub-FRT-CD2-FRT-Gal4, UAS-mCD8GFP stock. (b) Both the CSD neurons (asterisk) have been marked (bilateral flipout). (c, d) A small set of central neurons (arrowheads). Cell body of the CSDn is indicated by an asterisk in (c). Scale bar = 30 μm. [file 1749-8104-2-20-S1.jpeg]

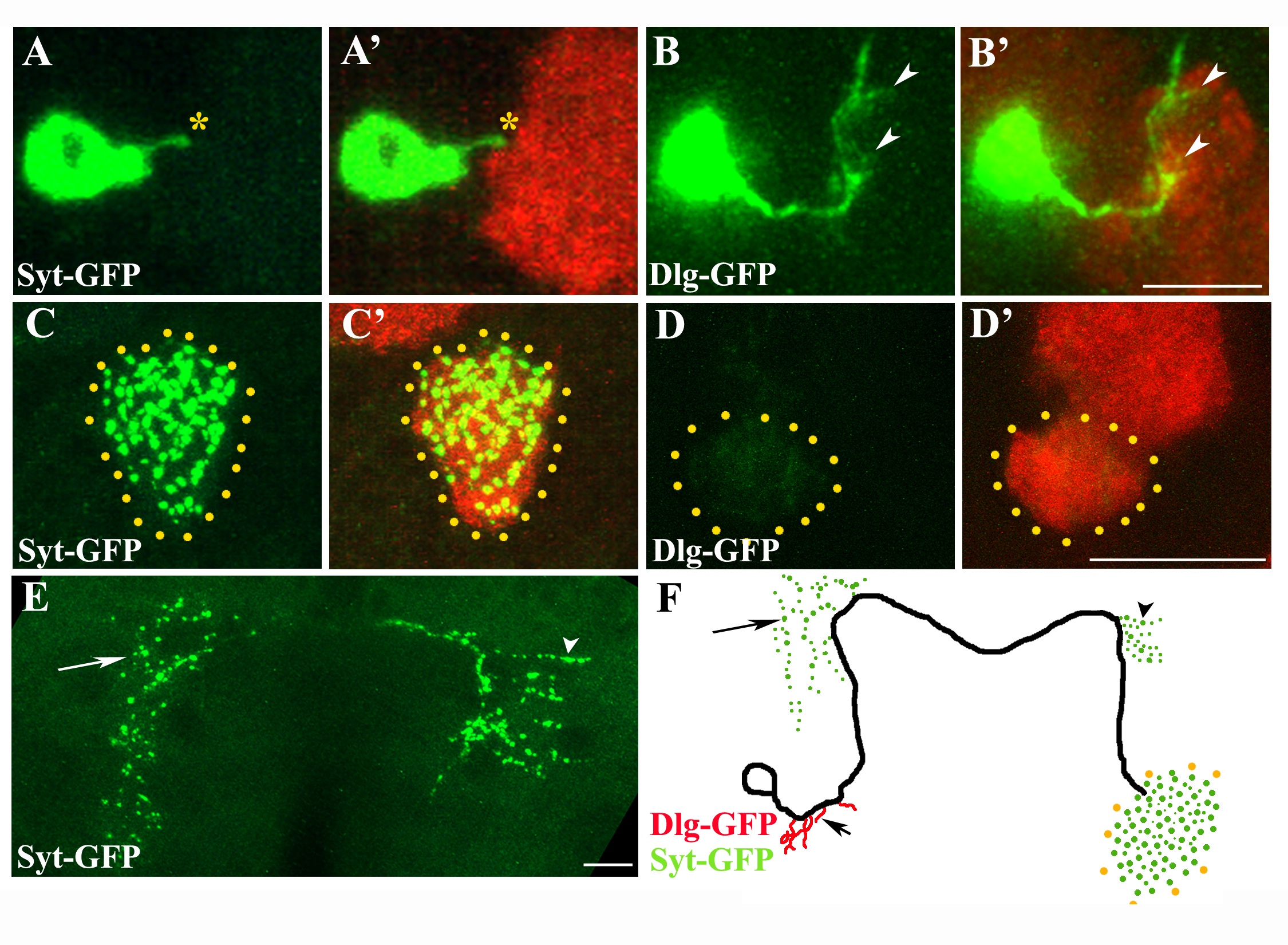

Supplement: Additional File 2 — Localization of Dlg-GFP and Syt-GFP in the larval CSD neuron. (a, c) RN2-Flp, Tub-FRT-CD2-FRT-Gal4/UAS-Syt-GFP. (b, d) UAS-Dlg-GFP/+; RN2-Flp, Tub-FRT-CD2-FRT-Gal4. (a, b) Ipsilateral dendrites; (c, d) contralateral presynaptic terminals stained with anti-GFP (green) and mAbnc82 (red). Syt-GFP localizes to the cell body but does not extend beyond a few microns in the primary neurite (asterisk; (a, a')), but is enriched within the synaptic endings in the antennal lobe (dotted lines) appearing as punctate staining (c, c'). Dlg-GFP is localized mainly within the cell body and ipsilateral neurites (arrowheads in (b, b') and is very weakly present in the terminal arbors within the antennal lobe (dotted lines in (d, d'). (e, e') Syt-GFP shows punctate localization in the branches at ipsilateral (arrow) and contralateral higher centers (arrowhead in (e, e'). (f) Diagrammatic representation of Syt-GFP (green) and Dlg-GFP (red) expression. Dlg-GFP is enriched in the dendritic trees while Syt-GFP is localized to the presynaptic terminals of CSDn. [file 1749-8104-2-20-S2.jpeg]

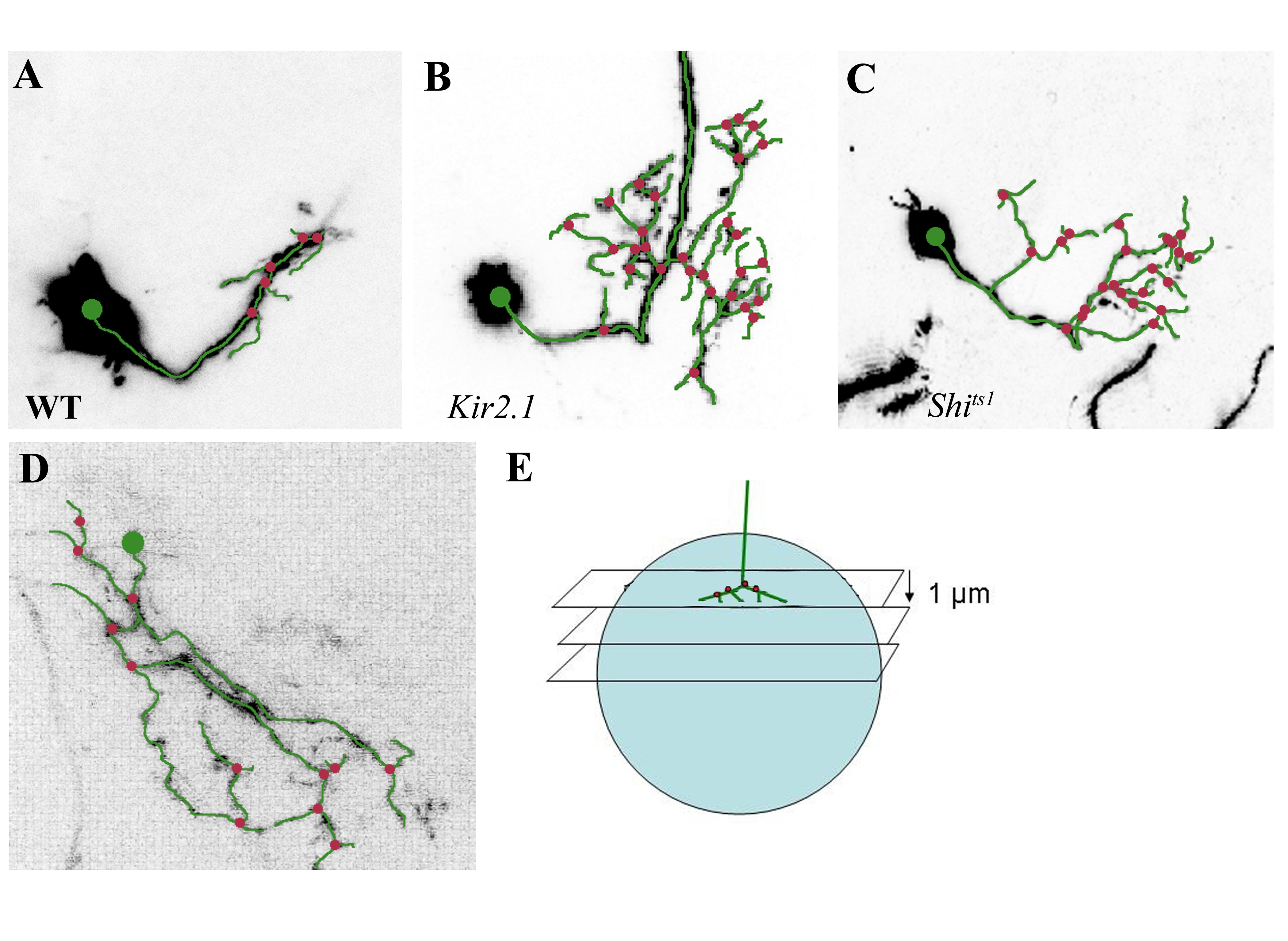

Supplement: Additional File 3 — Quantification of branch points. (a-d) Schematic of the branch point analysis of dendrites within the ipsilateral antennal lobe (a-c) and the contralateral terminals (d). (a, d) RN2-Flp, Tub-FRT-CD2-FRT-Gal4, UAS-mCD8GFP/+ dendritic (a) and terminal (d) arbors. (b) RN2-Flp, Tub-FRT-CD2-FRT-Gal4, UAS-mCD8GFP/UAS-EGFPKir2.1.(c) UAS-shits1/+; RN2-Flp, Tub-FRT-CD2-FRT-Gal4, UAS-CD8GFP/UAS-shits1. (e) Schematic showing that the neuron was imaged in 1 μm confocal sections through the brain. Each section was projected onto a monitor and the branch point denoted by a red dot on a cellulose acetate transparent sheet. The total number of branch points was counted over the whole z-stack. [file 1749-8104-2-20-S3.jpeg]
